# Musculoskeletal pain and re-employment among unemployed job seekers: a three-year follow-up study

**DOI:** 10.1186/s12889-016-3200-0

**Published:** 2016-07-08

**Authors:** Chioma A. Nwaru, Clas-Håkan Nygård, Pekka Virtanen

**Affiliations:** School of Health Sciences, University of Tampere, FI-33014 Tampere, Finland

**Keywords:** Unemployment, Musculoskeletal pain, Localized pain, Multiple pain sites, Re-employment

## Abstract

**Background:**

Poor health is a potential risk factor for not finding employment among unemployed individuals. We investigated the associations between localized and multiple-site musculoskeletal pain and re-employment in a three-year follow-up of unemployed job seekers.

**Methods:**

Unemployed people (*n* = 539) from six localities in southern Finland who participated in various active labour market policy measures at baseline in 2002/2003 were recruited into a three-year health service intervention trial. A questionnaire was used to collect data on musculoskeletal health and background characteristics at baseline and on employment status at the end of the follow-up. We conducted a complete case (*n* = 284) and multiple imputation analyses using logistic regression to investigate the association between baseline musculoskeletal pain and re-employment after three years.

**Results:**

Participants with severe pain in the lower back were less likely to become re-employed. This was independent of potential confounding variables. Pain in the hands/upper extremities, neck/shoulders, lower extremities, as well as multiple site were not determinants of re-employment.

**Conclusions:**

Our findings lend some support to the hypothesis that poor health can potentially cause health selection into employment. There is the need to disentangle health problems in order to clearly appreciate their putative impact on employment. This will allow for more targeted interventions for the unemployed.

**Electronic supplementary material:**

The online version of this article (doi:10.1186/s12889-016-3200-0) contains supplementary material, which is available to authorized users.

## Background

Unemployment has a detrimental effect on the health and well-being of individuals [1], their spouses [2], their children [3, 4], and the public at large [5–7]. Prospective studies have shown that re-employment could improve the health of the unemployed. Evidence of such improvement has been demonstrated in both a five-year [8] and a ten-year [9] follow-up study, where a significant improvement in mental health was reported among the unemployed after they re-entered paid employment. Schuring et al. [10] and Carlier et al. [11] also demonstrated that re-employment improved physical health, hence suggested that labour force participation should be considered as a therapeutic measure within the health promotion framework for the unemployed.

Poor health is an important risk factor for not finding employment. According to the health selection theory, unemployed persons with poor health may be less likely hired by prospective employers, thus are at risk of being selected into prolonged spell of unemployment [8, 12]. Many studies have investigated health selection using mental or physical health as determining factors. Findings regarding mental health are inconsistent. In a two-year follow-up study in Norway, mental disorders and physician-diagnosed psychiatric syndromes or personality disorders were risk factors for not regaining employment among long-term (more than 12 weeks) unemployed people [13]. In a five-year follow-up of that study, only the doctor’s diagnosis of psychiatric syndromes or personality disorders was however significantly associated with reduced re-employment [8]. In a three-year study in Finland, psychological distress was not associated with re-employment among registered unemployed persons [14], but a twelve-year follow-up study in Britain reported an increased likelihood of re-employment among unemployed women with psychological distress [15].

Regarding physical health, van de Mheen et al. [16] reported that poor general health, a chronic condition, and health complaints were determinants of re-employment after 4.5 years. Similar findings were reported in the European Household survey, with poor health and chronic conditions as determinants of not entering paid employment in most European countries [17]. Poor general health [18, 19] and decreased work performance due to impaired health [20] have also been shown to reduce likelihood of re-employment. One limitation in these studies is that the indicators of physical health were measured in a general context, i.e. in terms of chronic health problems or general self-rated health, which despite being important and valid measures – do not give indication of the specific roles of the health problems and diseases.

Musculoskeletal pain is a widespread problem among the working population, and it is a known risk factor for poor work ability [21, 22], increased absence due to sickness [23], early retirement [24], and health-related job loss [25]. Musculoskeletal pain may also reduce the possibility of regaining employment, but the evidence emerge from studies conducted among persons with arthritis and musculoskeletal disorders who were unemployed [26] and those of pre-retirement age [27]. Generalizing these findings to the general unemployed population would require further studies among individuals with differential symptom patterns and unemployment histories. In the present study, we investigate whether localized and multiple-site musculoskeletal pain are associated with re-employment in a three-year follow-up of registered unemployed people aged 18 to 59 years in Finland.

## Methods

### Study design and subjects

**Fig. 1:**
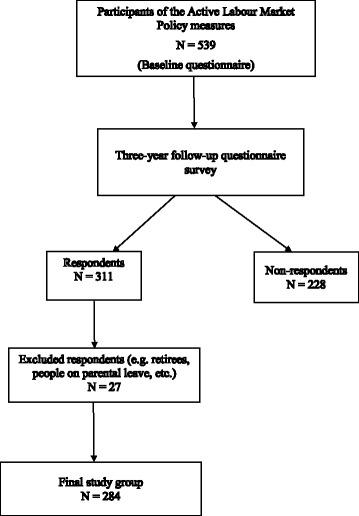
Flowchart of the participants’ response at baseline and follow-up

The study data originated from the Career Health Care (CHC) project, a three-year intervention trial that was launched in 2002–2003 in Finland with the goal of tackling health problems and risks related to unemployment [28]. Participants in the project were unemployed people (*n* = 539) from six localities in southern Finland who were enrolled in active labour market policy (ALMP) measures. They were recruited at the beginning of the ALMP measures, during which they received oral and written information about the study. This information made it explicit that participation was voluntary and not a condition for participation in the ALMP or access to the associated benefits. Those who consented to the study were randomly allocated to the intervention and control groups. The intervention group (*n* = 265) were recipients of the CHC package (i.e. the extra health services that targeted the unemployed). The control group (*n* = 274) only used communal health services. Both groups completed the baseline questionnaire during the recruitment exercise. Follow-up data was collected three years after the first encounter and 311 persons responded to this follow-up. The intervention group completed the follow-up questionnaires during the CHC encounter, and the control group returned their questionnaires by post. We excluded a group of respondents (*n* = 27) who were classified as non-job seekers at follow-up from the present study, because they were not at risk for unemployment. This gave rise to a sample of 284 people aged 18–59 years who responded to the three-year follow-up (see Fig. 1).

### Measurements

We measured musculoskeletal complaints at baseline using a modified version of the Nordic Musculoskeletal Questionnaire [29]. Respondents were asked to report, on a scale of 0 to 10, whether they had experienced pain or numbness in four locations during the preceding week. The locations were the hands or upper extremities, neck or shoulders, lower back, and the feet or lower extremities. The response for each pain variable was categorized into three groups: 0 = no pain, 1–5 = mild pain, and 6–10 = severe pain. To construct a multiple site pain measure, mild and severe categories were combined into any pain = 1 and no pain = 0. All four musculoskeletal pain variables were then added up and the summed variable was expressed as the number of sites with pain (from 0 = no pain in any site to 4 = pain in four sites).

Other variables that were measured at baseline and considered as potential confounders included age, gender, educational attainment, marital status, duration of unemployment, alcohol use, smoking, physical activity, somatic diseases, and depression. Age was categorized into three groups: “18–29”, “30–44”, and “45–59”. Educational attainment was classified into three levels: “college/university degree”, “vocational school degree”, and “no occupational degree”. Marital status was categorized as “single”, “married/cohabiting”, or “widowed/divorced”. Duration of unemployment was dichotomized to “less than one year” and “more than one year”. Alcohol use was elicited with the question “how often do you drink beer, wine or other alcoholic drinks?” The response was categorized into three: “never/less often”, “2–4 times a month”, and “2 or more times/week”. Smoking was dichotomized to “smokers” and “none smokers”, and leisure-time physical activity (i.e. frequency of vigorous physical activity for at least 15 to 20 min) was categorized into three: “not at all or only a little”, “moderate” (once per week), and “much” (twice or more per week). General health was assessed with the question “do you have diseases diagnosed by a physician?” A list of 18 different diseases was provided with a dichotomized reply (yes or no). We considered the responses that included one or more of the nine somatic diseases listed, i.e. cardiovascular illnesses, respiratory illnesses, diabetes, etc. (with the exception of musculoskeletal diseases). The sum score of the diseases was dichotomized (yes or no), and those subjects reporting one or more diseases were categorized as having somatic disease(s). Depression was measured with the Beck Depression Inventory [30] and dichotomized to “depressed” and “not depressed”.

Current employment status was determined in the three-year follow-up questionnaire and classified into two categories: “re-employed” and “unemployed”. Subjects were defined as re-employed if they reported being either employed or self-employed. The unemployed group consisted of those who reported not being in any paid job but seeking employment during the follow-up.

### Statistical analysis

The description of the subjects’ characteristics are presented as frequencies and percentages, and differences between groups were tested with a chi-squared test for categorical variables. The association between musculoskeletal pain and re-employment was examined with binary logistic regression. Re-employment was coded in such a manner that an odds ratio > 1 indicated an increased likelihood of re-employment. We conducted both complete-case (i.e. those who participated in both baseline and there-year follow-up) and multiple imputation (i.e. to impute data of the three-year follow-up for those who did not participate in the follow-up) analyses. The complete-case analysis was undertaken using IBM SPSS Statistics for Windows, version 20.0. (Armonk, NY: IBM Corp). In the complete-case analysis, unadjusted and adjusted models were performed. The unadjusted model (Model I) estimated the independent effect of the various localized pains, as well as the number of pain sites. The adjusted models included potential confounders in the model, with Model II simultaneously controlling for age, gender, educational attainment, and marital status. Model III additionally adjusted for the duration of unemployment, alcohol use, smoking, physical activity, somatic diseases, depression and participation in CHC. Although a recent study by Romppainen et al. (2014) did not find any beneficial effect of the CHC on re-employment, we also explored its role as a potential effect-modifying variable by entering an interaction term between musculoskeletal pain and participation in CHC in the adjusted model in relation to re-employment. If the interaction term was significant, we stratified the analysis by participation in CHC and calculated the stratum-specific estimates adjusting for all other confounders.

The multiple imputation (assuming missing at random) was conducted using the Multiple Imputation by Chained Equations (MICE) algorithm in Stata (version 13). A total of 20 imputed datasets were created. All variables that were used in the complete-case analysis, irrespective of whether they had missing or not, were included in the imputation model. After the imputation, we then repeated the Model III logistic regression analysis conducted with the complete-case analysis. An interaction term between musculoskeletal pain and participation in CHC was also investigated in the Model III of the multiple imputation model. Results are presented as odds ratios (OR) with their 95 % confidence intervals (CI), and their statistical significance was defined as the two-sided *p*-value <0.05.

## Results

At the three-year follow-up, 311 of the original 539 participants responded to the questionnaire survey (response rate 58 %). An analysis of non-respondents versus respondents showed a lower response rate among males (47 %) than among females (64 %), among smokers (50 %) than among none smokers (64 %), and among participants in the intervention (49 %) than among the control (66 %) group. Participants who were either widowed or divorced had the lowest response rate (47 %) compared to their counterparts who were single (52 %) or were married or cohabiting (64 %). Differences in other individual characteristics (age, educational attainment, alcohol use, physical activity, somatic diseases, and depression) as well as musculoskeletal pain were not statistically significant (see Additional file 1: Table S1).

By excluding 27 (9 % of those who completed both questionnaire) ineligible respondents, who consisted of retirees or those receiving disability pension (*n* = 9), those on parental leave (*n* = 7), non-job seekers (*n* = 1), or those excluded for some other reason (*n* = 10), the subsequent analyses included 52 % (284/539) of the original study population. The baseline individual and health characteristics of the 284 respondents are given in Table 1. The participants were predominantly middle-aged (45 %, *n* = 127), with most of them (67 %, *n* = 190) having been unemployed for less than one year at baseline. Twenty-two percent of them had attained a college/university degree. In the week preceding the baseline measurement, 147 (52 %) reported mild-to-severe pain in the hands/upper extremities, 195 (69 %) in the neck/shoulders, 154 (52 %) in the lower back, and 141 (50 %) in the feet/lower extremities. Over half of the respondents (59 %, *n* = 168) had concurrent pain in two or more sites.

**Table 1:** Distribution of study participants by baseline socio-demographic and health characteristics

	Unemployed job-seekers (*N* = 284)
Individual characteristics	*n* (%)
Age (years)	
18–29	68 (23.9)
30–44	127 (44.7)
45–59	80 (28.2)
Missing	9 (3.2)
Gender	
Male	89 (31.3)
Female	194 (68.3)
Missing	1 (0.4)
Educational attainment	
No occupational education	93 (32.7)
Vocational school	120 (42.3)
College/university	64 (22.5)
Missing	7 (2.5)
Marital status	
Single	82 (28.9)
Married/cohabiting	170 (59.9)
Widowed/divorced	30 (10.6)
Missing	2 (0.7)
Duration of unemployment	
Less than one year	190 (66.9)
More than one year	94 (33.1)
Participation in CHC	
Intervention group	119 (41.9)
Control group	165 (58.1)
Lifestyle/health characteristics	
Alcohol use	
Never/less often	113 (39.8)
2–4 times/month	128 (45.1)
2 or more times/week	43 (15.1)
Smoker	
No	179 (63.0)
Yes	105 (37.0)
Physical activity	
Much	91 (32.0)
Moderate	70 (24.6)
Not at all or only a little	111 (39.1)
Missing	12 (4.2)
Somatic diseases	
No	153 (53.9)
Yes	110 (38.7)
Missing	21 (7.4)
Depression	
No	253 (89.1)
Yes	17 (6.0)
Missing	14 (4.9)
Hands/upper extremity pain	
None	120 (42.3)
Mild	90 (31.7)
Severe	57 (20.1)
Missing	17 (6.0)
Neck/shoulder pain	
None	75 (26.4)
Mild	119 (41.9)
Severe	76 (26.8)
Missing	14 (4.9)
Low back pain	
None	106 (37.3)
Mild	106 (37.3)
Severe	48 (16.9)
Missing	24 (8.5)
Feet/lower extremity pain	
None	126 (44.4)
Mild	96 (33.8)
Severe	45 (15.8)
Missing	17 (6.0)
Number of musculoskeletal pain sites	
0	74 (26.1)
1	42 (14.8)
2	46 (16.2)
3	50 (17.6)
4	72 (25.4)

Participants with somatic diseases were more likely to report pain compared to those without somatic diseases, regardless of the pain type (Table 2). Reporting pain also increased with decreasing participation in vigorous physical activity although the differences were significant only for low back pain (*p* = 0.016) and lower extremity pain (*p* = 0.047). Other characteristics, such as age, gender, educational attainment, marital status, duration of unemployment, participation in CHC, alcohol use, smoking, and depression were not significant determinants of most musculoskeletal pain. Regarding employment status during the three-year follow-up, over half (55 %, *n* = 156) of the participants were re-employed. The likelihood of re-employment decreased with increasing age and decreasing educational attainment. Participants who were either widowed or divorced (40 %) were less likely to regain employment than those who were either single (49 %) or married/cohabiting (61 %).

**Table 2:** Individual characteristics of participants by baseline musculoskeletal pain and re-employment at three-year follow-up

	Baseline musculoskeletal pain									
	% with no hands/upper extremity pain	*p*-value	% with no neck/shoulder pain	*p*-value	% with no low back pain	*p*-value	% with no feet/lower extremity pain	*p*-value	% re-employed at 3-year follow-up	*p*-value
	(*n* = 120)		(*n* = 75)		(*n* =106)		(*n* = 126)		(*n* = 156)	
Age (years)		0.079		0.410		0.420		0.605		0.001
18–29	54.5		25.8		43.3		53.0		67.6	
30–44	46.3		30.3		44.4		49.6		59.1	
45–59	37.5		27.4		35.3		40.3		38.8	
Gender		0.819		0.001		0.286		0.342		0.222
Male	44.8		42.4		47.6		41.4		49.4	
Female	45.3		20.7		37.7		50.3		57.2	
Educational attainment		0.098		0.001		0.167		0.119		0.051
No occupational educ.	40.9		25.8		38.8		44.3		46.2	
Vocational educ.	43.6		24.3		36.8		45.5		56.7	
College/university	54.0		35.9		54.8		58.1		65.6	
Marital status		0.023		0.702		0.957		0.174		0.046
Single	38.0		27.5		42.3		39.2		48.8	
Married/cohabiting	50.3		26.5		39.4		53.1		60.6	
Widowed/divorced	35.7		34.6		46.2		37.0		40.0	
Duration of unemployment		0.970		0.081		0.616		0.745		0.093
Less than one year	45.2		24.2		39.4		47.2		58.4	
More than one year	44.4		35.2		43.5		47.2		47.9	
Participation in CHC		0.645		0.898		0.124		0.163		0.929
Intervention group	43.0		26.3		38.8		40.9		54.6	
Control group	46.4		28.8		41.4		52.0		55.2	
Alcohol use		0.692		0.363		0.432		0.456		0.879
Never/less often	48.1		25.7		45.6		43.4		53.1	
2–4 times/month	43.8		25.4		34.5		46.7		56.3	
2 or more times/week	40.5		39.5		46.3		58.5		55.8	
Smoker		0.819		0.965		0.442		0.028		0.679
No	43.9		27.2		38.0		44.8		55.9	
Yes	46.6		28.7		45.4		51.0		53.3	
Physical activity		0.128		0.068		0.016		0.047		0.774
Much	55.4		35.6		53.6		55.3		54.9	
Moderate	41.8		24.3		36.4		50.7		58.6	
Not at all/only a little	39.0		20.0		32.3		36.5		53.2	
Somatic diseases		0.006		0.057		0.002		0.001		0.118
No	52.8		32.7		50.0		54.5		58.8	
Yes	32.7		19.2		29.3		35.2		49.1	
Depression		0.059		0.063		0.220		0.007		0.082
No	47.1		29.3		43.0		49.8		56.9	
Yes	17.6		17.6		23.5		17.6		35.3	

*P*-value by *χ* ^2^ tests

Table 3 shows the results of the associations between musculoskeletal pain at baseline and re-employment after three years. Based on the unadjusted result, those with severe pain in the lower back or feet/lower extremities had a reduction of up to 59 % in the likelihood of re-employment. In the adjusted models, the reduced likelihood of re-employment with pain in the lower back (OR 0.37, 95 % CI 0.15–0.92) or feet/lower extremities (OR 0.38, 95 % CI 0.15–0.93) remained unchanged even after controlling for age, gender, educational attainment, marital status, duration of unemployment, participation in CHC, alcohol use, smoking, physical activity, somatic diseases, and depression. A reduced likelihood for re-employment was also found for those participants with three (OR 0.48, 95 % CI 0.23–0.99) or four (OR 0.51, 95 % CI 0.27–0.99) pain sites, although these associations were not retained when adjustments for confounders were introduced into the model (Table 4). The interaction between participation in CHC and musculoskeletal pain was not significant for most pain types except for low back pain. When we stratified the analysis by participation in CHC, the estimated odds for finding employment was significantly lower for those individuals in the control group who had severe low back (OR 0.18, 95 % CI 0.04–0.77) (see Additional file 2: Table S2).

**Table 3:** Associations between localized pain at baseline and re-employment at three-year follow-up

Localized musculoskeletal pain	Re-employment at 3-year follow-up			
	OR (95 % CI)			
	Model I^a^	Model II^b^	Model III^c^	Multiple imputation model^d^
Hands/upper extremity				
None	1.00	1.00	1.00	1.00
Mild	0.84 (0.48–1.47)	1.45 (0.76–2.73)	1.40 (0.69–2.87)	1.22 (0.67–2.20)
Severe	0.54 (0.28–1.02)	0.63 (0.31–1.27)	0.63 (0.28–1.38)	0.54 (0.27–1.09)
Neck/shoulder				
None	1.00	1.00	1.00	1.00
Mild	0.93 (0.51–1.66)	1.01 (0.52–1.94)	0.87 (0.42–1.81)	1.05 (0.50–2.23)
Severe	0.78 (0.41–1.49)	0.72 (0.35–1.49)	0.99 (0.44–2.24)	0.72 (0.41–2.32)
Low back				
None	1.00	1.00	1.00	1.00
Mild	0.92 (0.53–1.60)	1.11 (0.61–2.04)	0.96 (0.48–1.90)	1.07 (0.50–2.29)
Severe	0.41 (0.21–0.83)	0.40 (0.18–0.88)	0.37 (0.15–0.92)	0.35 (0.16–0.78)
Feet/lower extremity				
None	1.00	1.00	1.00	1.00
Mild	0.73 (0.42–1.25)	1.10 (0.60–2.01)	1.20 (0.60–2.40)	1.05 (0.48–2.29)
Severe	0.41 (0.20–0.82)	0.46 (0.21–0.98)	0.38 (0.15–0.93)	0.51 (0.22–1.16)

^a^Unadjusted model

^b^Adjusted for age, gender, educational attainment, and marital status

^c^Adjusted Model II + duration of unemployment, participation in CHC, alcohol use, smoking, physical activity, somatic diseases and depression

Models I, II, and III are based on complete-case analysis (*N* = 284)

^d^Adjusted for age, gender, educational attainment, marital status, duration of unemployment, participation in CHC, alcohol use, smoking, physical activity, somatic diseases and depression (*N* = 539)

**Table 4:** Associations between number of musculoskeletal pain sites at baseline and re-employment at three-year follow-up

Number of musculoskeletal pain sites	Re-employment at 3-year follow-up			
	OR (95 % CI)			
	Model I^a^	Model II^b^	Model III^c^	Multiple imputation model^d^
0	1.00	1.00	1.00	1.00
1	0.99 (0.45–2.16)	0.82 (0.36–1.88)	0.85 (0.35–2.10)	0.86 (0.40–1.84)
2	1.04 (0.48–2.22)	1.26 (0.55–2.88)	1.57 (0.61–4.02)	1.05 (0.48–2.27)
3	0.48 (0.23–0.99)	0.58 (0.26–1.29)	0.86 (0.35–2.09)	0.68 (0.31–1.47)
4	0.51 (0.27–0.99)	0.72 (0.35–1.49)	0.69 (0.29–1.61)	0.66 (0.33–1.32)

^a^Unadjusted model

^b^Adjusted for age, gender, educational attainment, and marital status

^c^Adjusted Model II + duration of unemployment, participation in CHC, alcohol use, smoking, physical activity, somatic diseases and depression

Models I, II, and III are based on complete-case analysis (*N* = 284)

^d^Adjusted for age, gender, educational attainment, marital status, duration of unemployment, participation in CHC, alcohol use, smoking, physical activity, somatic diseases and depression (*N* = 539)

Results from the complete-case and multiple imputation analyses were generally similar to each other, except that the confidence interval for lower extremity pain included one in the multiple imputation analysis (complete-case: OR 0.38, 95 % CI 0.15–0.93; multiple imputation: OR 0.51, 95 % CI 0.22–1.16). In addition, the significant interaction effect between low back pain and participation in CHC observed in the complete-case analysis was not seen in the multiple imputation analysis, suggesting that the complete-case interaction may be a chance finding.

## Discussion

We found that severe pain in the lower back was associated with a reduced likelihood of re-employment after three years among unemployed job seekers. Pain in the hands/upper extremities, neck/shoulders, the lower extremities, as well as multiple site did not influence re-employment. These results were similar both in complete-case and multiple imputation analyses.

We recorded a moderate but acceptable participation rate of 58 % at three-year follow-up, which is similar to those achieved in previous studies [31, 32]. Usually high drop-out rates have been observed for the unemployed [33, 34]. Although differences between participants and non-participant at the three-year follow-up were observed only for sex, marital status, smoking, and participation in the CHC, we undertook multiple imputation analysis to impute missing data for those who did not take part in the follow-up assessment. This provided us with relevant sensitivity analysis to appraise the extent of bias due to follow-up with the complete-case analysis. Our assessment of the subjects’ musculoskeletal pain status was based on a self-report, which may introduce information bias, however self-reporting of pain indicators has been noted to be reliable [29] and it is commonly used for pain studies [24, 35, 36]. The time into the past (one week) participants were asked to recall any pain is short and therefore should minimize the risk of recall bias.

There may be the possibility of residual confounding since we could not assess the influence of all potential confounders, particularly body mass index, although previous studies [37] did not find an independent association between body mass index and re-employment. The generalizability of our findings is equally limited owing to the fact that our data were based on unemployed persons who actively participated in various labour market policy measures. Hence, they constituted a relatively unique group that may not be representative of the unemployed population as a whole. Nonetheless, the findings of this study reflect evidence from unemployed people who still belong to the labour force. Vesalainen and Vuori [14] showed that the level of job-seeking activities might influence an individual’s probability of finding a job. It is also possible that the level of job-seeking activities will vary among members of different unemployment groups. Our study excluded those in other unemployment groups such as retirees, those receiving disability pensions, those on parental leave, non-job seekers, and those in other situations who are likely to adopt passive job-seeking behaviour.

Our findings of reduced re-employment among participants with severe lower back pain supports those of Straaton et al. [26], Yelin, Trupin & Sebasta [27], and Virtanen, Janlert, & Hammarstöm [37], who all showed that musculoskeletal pain was a determinant factor in regaining re-employment. The contribution of the present study is that it distinguished pain in local sites from that in multiple sites, and provided insight into their respective roles in the relationship between health and employment. This is necessary considering that the differences in the risk factors and prognosis of the various pain types require different interventional measures.

A potential explanation why pain in the lower back was associated with a reduced likelihood of re-employment while pain in the other body regions (hands/upper extremities, neck/shoulders, and lower extremities) was not may be that low back pain may have persisted during periods of unemployment and thus, discouraged the motivation for finding employment. The occurrence of low back pain is not only associated with work-related factors, but also with psychological (anxiety, depression, emotional instability) and lifestyle-related (smoking and excess body weight) factors [38], which are prevalent among unemployed individuals [39, 40]. In addition, empirical evidence has shown that pain in the lower back is highly recurrent and rarely resolves [38], with some studies showing that low back pain may be associated with activity restriction [41]. It could be that these characteristics of low back pain may limit job search activities among individuals suffering from severe low back pain.

It was a surprising finding that the number of pain sites was not associated with re-employment considering the deleterious impact of pain on work and productivity [24, 42]. It is possible that pain in multiple sites is less burdensome during periods of unemployment due to reduced exposure to occupational factors that are considered major predisposing agents for pain in multiple sites [43].

## Conclusion

In this study, we find that severe low back pain is a significant determinant of re-employment among unemployed job-seekers. This finding demonstrates the need to disentangle health problems in order to clearly appreciate their putative impact on employment. This is of paramount importance, especially for those health problems that may be modifiable. In further research, it would be helpful to understand whether similar associations may exist for chronic versus acute musculoskeletal pain.

## Abbreviations

CHC, Career Health Care; ALMP, Active labour market policy; MICE, Multiple Imputation by Chained Equations

## Additional files

Additional file 1: Table S1. Relation of background characteristics to respondents to the questionnaire survey at 3-year follow-up. (DOCX 18 kb) Additional file 2: Table S2. Modification of the effect of low back pain on re-employment by participation in CHC. (DOCX 16 kb)
