# Supplementary material for: Musculoskeletal pain and re-employment among unemployed job seekers: a three-year follow-up study
Source: BMC Public Health. 2016 Jul 8;16:531. doi: 10.1186/s12889-016-3200-0 (PMC4938954; doi:10.1186/s12889-016-3200-0)
Supplement: Additional file 1: Table S1. — Relation of background characteristics to respondents to the questionnaire survey at 3-year follow-up. (DOCX 18 kb) [file 12889_2016_3200_MOESM1_ESM.docx]

Additional file 1: Relation of background characteristics to respondents to the questionnaire survey at 3-year follow-up

|  | Total study population  N = 539  n (%) | Respondents at 3-year follow-up | P-value |
| --- | --- | --- | --- |
| Individual characteristics |  |  |  |
| Age (years)  18-29  30-44  45-59  Missing | 153 (28.4)  233 (43.2)  140 (26.0)  13 (2.4) | 78 (51.0)  134 (57.5)  90 (64.3) | 0.071 |
| Gender  Male  Female  Missing | 204 (37.8)  333 (61.8)  2 (0.4) | 97 (47.5)  213 (64.0) | 0.000 |
| Educational attainment  No occupational education  Vocational school  College/university  Missing | 202 (37.5)  225 (41.7)  102 (18.9)  10 (1.9) | 107 (53.0)  130 (57.8)  67 (65.7) | 0.105 |
| Marital status  Single  Married/cohabiting  Widowed/divorced  Missing | 178 (33.0)  294 (54.5)  64 (11.9)  3 (0.6) | 92 (51.7)  187 (63.6)  30 (46.9) | 0.007 |
| Duration of unemployment  Less than one year  More than one year | 360 (66.8)  179 (33.2) | 209 (58.1)  102 (57.0) | 0.812 |
| Participation in CHC  Intervention group  Control group | 265 (49.2)  274 (50.8) | 130 (49.1)  181 (66.1) | 0.000 |
| Lifestyle/ health characteristics |  |  |  |
| Alcohol use  Never/ less often  2-4 times/month  2 or more times/week  Missing | 223 (41.4)  236 (43.8)  79 (14.7)  1 (0.2) | 127 (57)  135 (57)  49 (62) | 0.712 |
| Smoker  No  Yes | 309 (57.3)  230 (42.7) | 197 (63.8)  114 (49.6) | 0.001 |
| Physical activity  Much  Moderate  Not at all or only a little  Missing | 179 (33.2)  122 (22.6)  217 (40.1)  21(3.9) | 101 (56.4)  75 (61.5)  121 (55.8) | 0.567 |
| Somatic diseases  No  Yes  Missing | 284 (52.7)  206 (38.2)  49 (9.1) | 169 (59.5)  120 (58.3) | 0.780 |

Additional file 1: Continued

| Depression  No  Yes  Missing | 466 (86.5)  42 (7.8)  31 (5.8) | 274 (58.8)  21 (50.0) | 0.268 |
| --- | --- | --- | --- |
| Hands/upper extremity pain  No  Mild  Severe  Missing | 225 (41.7)  162 (30.1)  117 (21.7)  35 (6.5) | 130 (57.8)  95 (58.6)  69 (59.0) | 0.973 |
| Neck/shoulder pain  No  Mild  Severe  Missing | 144 (26.7)  216 (40.1)  150 (27.8)  29 (5.4) | 84 (58.3)  125 (57.9)  88 (58.7) | 0.988 |
| Low back pain  No  Mild  Severe  Missing | 204 (37.8)  196 (36.4)  90 (16.7)  49 (9.1) | 117 (57.4)  110 (56.1)  58 (64.4 | 0.397 |
| Feet/lower extremity pain  No  Mild  Severe  Missing | 241 (44.7)  174 (32.3)  86 (16.0)  38 (7.1) | 139 (57.7)  100 (57.5)  54 (62.8) | 0.672 |
| Number of musculoskeletal pain sites  0  1  2  3  4 | 137 (25.4)  85 (15.8)  96 (17.8)  86 (16.0)  135 (25.0) | 83 (60.6)  46 (54.1)  47 (49.0)  54 (62.8)  81 (60.0) | 0.275 |
