# Supplementary material for: Musculoskeletal pain and re-employment among unemployed job seekers: a three-year follow-up study
Source: BMC Public Health. 2016 Jul 8;16:531. doi: 10.1186/s12889-016-3200-0 (PMC4938954; doi:10.1186/s12889-016-3200-0)
Supplement: Additional file 2: Table S2. — Modification of the effect of low back pain on re-employment by participation in CHC. (DOCX 16 kb) [file 12889_2016_3200_MOESM2_ESM.docx]

Additional file 2: Modification of the effect of low back pain on re-employment by participation in CHC

|  | Participation in CHC | |
| --- | --- | --- |
|  | Intervention group  OR (95%CI) | Control group  OR (95%CI) |
| Low back pain |  |  |
| No  Mild  Severe | 1.00  0.49 (0.15-1.64)  0.69 (0.16-3.00) | 1.00  0.16 (0.64-4.24)  0.18 (0.04-0.77) |

Adjusted for age, gender, educational attainment, marital status, duration of unemployment, alcohol use, smoking, physical activity, somatic diseases and depression
